# Editorial: Germinal Centers in Lymphoid and Non-Lymphoid Tissues: Adaptive and Evolving Structures

**DOI:** 10.3389/fimmu.2022.880733

**Published:** 2022-03-29

**Authors:** Michel Cogné, Thierry Fest, Said Aoufouchi

**Affiliations:** ^1^ University of Limoges, Limoges, France; ^2^ University of Rennes 1, Rennes, France; ^3^ INSERM UMR1236 Microenvironnement, Différenciation cellulaire, Immunologie et Cancer, Rennes, France; ^4^ UMR 9019 Intégrité du Génome et Cancers, Centre National de la Recherche Scientifique, Villejuif, France; ^5^ Université Paris-Saclay, Saint Aubin, France; ^6^ Institut Gustave Roussy, Villejuif, France

**Keywords:** germinal center (GC) B cell, affinity maturation, humoral immune response, Tertiary Lymphoid Structures (TLS), GC B cell outcomes, cell fate decision, B and T cell interactions, B cell lymphoma

Germinal centers (GC) are central places for the development of adaptive immune responses. While their development follows a first wave of extra-follicular activation, they uniquely contribute to the development of long-term responses including both diversified memory B cells with strong ability to activate and differentiate upon antigen re-challenge, and long-lived plasma cells producing high-affinity class-switched antibodies. They are central to protective immune responses against microbial or tumor-associated antigens, but they are also major sites where dealing with peripheral tolerance to autoantigens, programmed cell death and with the hazardous outcomes of DNA lesions inflicted to B-cells.

There is currently no definitive model accounting for all the outcomes of GC formation, since these evolving structures integrate stimulating and inhibitory signals from multiple origins, including antigens, chemokines, cytokines, specific antibody level and they dynamically evolve so that the very same factors initially promoting GC development can later contribute to GC resolution.

In contexts of chronic local inflammation, “GC-like” tertiary lymphoid structures (TLS) have long been reported within some non-lymphoid tissues and they recently received strong attention for their contribution to tumor immunology. They share structural and functional characteristics with GC that form in the secondary lymphoid organs. TLS are induced by persistent infection, autoimmune disorders and cancer. Interestingly in many cancer types, a good correlation has been reported between richness in TLS in tumor site and prolonged patient survival (1, Trüb et al.). The cellular and molecular signals that govern the induction and the fate of TLS in such pathological situations are not well-understood, but are among the hotest research questions in this emerging TLS-Cancer topic with potential for new discovery in B cell focused immunotherapies. We therefore believe that classical GC and TLS structures are the two sides of the general mechanism of immune response and immune surveillance.

In this Research Topic of Frontiers in Immunology, Kennedy and Clark from Chicago University, review the general compartments and connections at work in GCs. In a perspective paper notably commenting conflicting data about the role of hypoxia within the GC, Boothby et al. propose standards for analyzing and reporting data sets from GC cells differentiated under variable immunization constraints. This Research Topic additionally reports a multiscale model in which Tejero et al. combine the expected effects of both asymetric divisions of B cells, the network of their cellular interactions and the strength of affinity-based CD40-signalling for determining the outcome of B-cell activation towards either memory MBC) or plasma cells (PC). In this regard, Nakagawa and Calado review the conditions mediating positive selection of light zone(LZ) B-cells and instructing them to be selected as either PCs, MBCs or persistent GC-B cells reentering the dark zone. Santamaria et al. present new experimental data in human showing that this cell fate decision is also impacted by a threshold of IL-4/STAT6 signaling making GC LZ B cells either proliferate and transiently express a MYC-dependent transcriptional program or rather up-regulate BLIMP and instructively progress towards PC differentiation when CD23-dependent signaling becomes lower. Lemarié et al. also show induction of the unfolded protein response genes as a very early event at the pre-plasmablastic stage.

Going deeper into the diversity of cell interactions, Lu and Craft review recent evidence about the contribution of Tfr cells in the balance between productive immune responses and B cell memory and the maintenance of homeostasis for avoiding immunopathology.

Rivas et al., analyzing both murine models and data from human DLBCL patients, provide new evidence about the caretaker role of the cohesin complex in genomic stability of GC B-cells. They notably show that cohesin ATPase subunit Smc3 haploinsufficiency favors malignant transformation, through gene repression and impaired enhancer-promoter interactions, with loss of epigenetic modifiers (TET2 or KMT2D) preventing B-cell exit from the GC reaction and their commitment to plasma cell differentiation.

Schmiedel et al., using a Brg-1 deficient model, also provide data showing that ATPase, Brg1 and the BAF chromatin remodeling complex, are required for enhancer-promoter interactions which promote cell cycle-related gene expression during GC formation.

Dauba and Khamlichi review into much details the long -range promoter-enhancer interactions within the IgH locus which promotes chromatin remodeling and synapses between target switch sequences in a B-cell stimulation-dependent manner in order to support CSR. Dalloul et al. additionally provide new evidence that the process of Cclass switch recombination (CSR) can initiate even in the absence of the activation induced cytidine deaminase (AID) enzyme as an intrinsic ability of an appropriately conformed IgH locus to undergo recombination, so that AID is rather catalyzing and boosting CSR rather than initiating CSR in GC B-cells. Fuertes et al. finally review the role of multiple microRNAs (miRNAs) in the regulation of Tfh and GC B-cell responses as well as in B cell neoplasia and GC response dysregulation.

It altogether appears that cell fate decisions from GC B cells integrate both the quality and the cumulative amounts of signals that they have received from the Ag and their local microenvironment, following an instructive model, and their intrinsic ability to be committed to proliferation or differentiation, with cell death as another major fatal outcome of B cell activation and AID induction (2–4).

## Author Contributions

All authors listed have made a substantial, direct, and intellectual contribution to the work, and approved it for publication.

## Conflict of Interest

The authors declare that the research was conducted in the absence of any commercial or financial relationships that could be construed as a potential conflict of interest.

## Publisher’s Note

All claims expressed in this article are solely those of the authors and do not necessarily represent those of their affiliated organizations, or those of the publisher, the editors and the reviewers. Any product that may be evaluated in this article, or claim that may be made by its manufacturer, is not guaranteed or endorsed by the publisher.

## References

[1] Sautès-Fridman C Verneau J Sun C-M Moreira M Chen TW-W Meylan M. Tertiary Lymphoid Structures and B Cells: Clinical Impact and Therapeutic Modulation in Cancer. Semin Immunol (2020) 48:101406. doi: 10.1016/j.smim.2020.101406 33248905

[2] Péron S Laffleur B Denis-Lagache N Cook-Moreau J Tinguely A Delpy L. AID-Driven Deletion Causes Immunoglobulin Heavy Chain Locus Suicide Recombination in B Cells. Science (2012) 336:931–4. doi: 10.1126/science.1218692 22539552

[3] Dalloul I Boyer F Dalloul Z Pignarre A Caron G Fest T. Locus Suicide Recombination Actively Occurs on the Functionally Rearranged IgH Allele in B-Cells From Inflamed Human Lymphoid Tissues. PLoS Genet (2019) 15:e1007721. doi: 10.1371/journal.pgen.1007721 31199803 PMC6594652

[4] Mayer CT Gazumyan A Kara EE Gitlin AD Golijanin J Viant C. The Microanatomic Segregation of Selection by Apoptosis in the Germinal Center. Science (2017) 358. doi: 10.1126/science.aao2602 PMC5957278 28935768

